# Characterizing the International Migration Barriers with a Probabilistic Multilateral Migration Model

**DOI:** 10.1038/srep32522

**Published:** 2016-09-06

**Authors:** Xiaomeng Li, Hongzhong Xu, Jiawei Chen, Qinghua Chen, Jiang Zhang, Zengru Di

**Affiliations:** 1 School of Systems Science, Beijing Normal University Beijing 100875, P. R. China.

## Abstract

Human migration is responsible for forming modern civilization and has had an important influence on the development of various countries. There are many issues worth researching, and “the reason to move” is the most basic one. The concept of migration cost in the classical self-selection theory, which was introduced by Roy and Borjas, is useful. However, migration cost cannot address global migration because of the limitations of deterministic and bilateral choice. Following the idea of migration cost, this paper developed a new probabilistic multilateral migration model by introducing the Boltzmann factor from statistical physics. After characterizing the underlying mechanism or driving force of human mobility, we reveal some interesting facts that have provided a deeper understanding of international migration, such as the negative correlation between migration costs for emigrants and immigrants and a global classification with clear regional and economic characteristics, based on clustering of migration cost vectors. In addition, we deconstruct the migration barriers using regression analysis and find that the influencing factors are complicated but can be partly (12.5%) described by several macro indexes, such as the GDP growth of the destination country, the GNI per capita and the HDI of both the source and destination countries.

In this globalization wave, the scale and diversity of international migration are increasing sustainably^1^. “In 2013, globally, there were 232 million international migrants and 41% are hosted by the developing regions. … Between 1990 and 2013, the number of international migrants worldwide rose by over 77 million or by 50 percent, and much of this growth occurred between 2000 and 2010”. The 2013 U.N. International Migration Report reveals a rapid and unbalanced growth of global migration flows, which has garnered much attention in related fields.

Considering immigrants as the research object, much work has been performed in this field. Aside from research on migration patterns^2^,^3^,^4^,^5^,^6^, the effects and consequences of international migration have intrigued many researchers. Researchers have discussed the social and economic influence of migration on the origin and destination countries. Generally, the migration of skilled workers benefits destination countries, but the effect on the origin countries is controversial. Some researchers hold that migration is harmful to the origin countries^7^,^8^,^9^,^10^. However, other researchers have identified the potential benefits of brain drain or brain gain^11^,^12^,^13^,^14^,^15^,^16^,^17^,^18^,^19^. Although different opinions exist, most studies have acknowledged the importance of migration flows to both the origin and destination countries. Other works have focused on the complex effective factors of human migration. However, most studies use qualitative analyses without quantification. This is because migration is a complex field of research that includes topics from a wide range of fields, such as development, trade, fiscal studies, demography, policy, and human resources^20^. So far, the quantitative analysis and estimation of the factors affecting migration patterns have remained on the research frontier. The reasons for migration are basic and critical; establishing those reasons would help to explain the current circumstances of international migration, predict the evolution of migration patterns, and even design more beneficial migration policies.

In quantitative analysis and estimation research, the classical self-selection theory is a fundamental component. Roy, a pioneering researcher, proposed an explanation for people migrating between countries or regions by modelling the optimization choice between fishing and hunting^21^. Following his idea, Borjas developed a formal mathematical model called the self-selection theory^22^,^23^,^24^. Based on the individual rational choice of a potential emigrant, it shows that macro migration flows can be described by income gaps between countries. Self-selection theory successfully presents the concept of migration costs or barriers, which are integrated factors that have an impact on migrant decisions beyond income. This has introduced a series of subsequent studies. Some studies have analysed the performance of migrants in the host labour market and have attempted to either verify or reject the self-selection theory. Results from an empirical analysis of immigrants from Mexico to the U.S. are inconsistent with the negative-selection hypothesis^25^; Borjas examined low-skilled out-migrants and highly skilled in-migrants in Puerto Rico and verified the self-selection theory in the local area^26^. Some researchers have used data from the OECD to discuss the difference between the origin and destination countries that influences the immigrant quality and quantity. The fundamental is verified limitation of classical self-selection model in the same time^27^,^28^.

However, for this theory, there are still some controversies surrounding the estimating of the migration cost. Some researchers even doubt the concept of migration costs because there is no explicit method for quantifying the integrated factors, and this makes empirical analysis unfeasible. By contrast, the burgeoning literature on global migration has shown that labour mobility restrictions have rapidly reduced incoming migration flows to developed countries^29^,^30^,^31^. Some studies have attempted to use specific data to discuss the impact of mobility restrictions on migration flows in certain locations, such as the West Bank^32^,^33^,^34^. Adnan defines mobility restrictions as visa requirements, border patrols, and immigration policies, which include quotas, point-based systems, and border closures, and these are typical non-income factors. Adnan uses survey and official data to analyse the significance of migration costs in the West Bank^34^. It is insufficient to simply analyse the migration costs regionally. However, there are no generally accepted standards to quantify the migration costs globally because the costs include complex integrated factors.

Aside from the difficulty of directly calculating the migration costs, there are other limitations to the traditional self-selection theory. Here, individuals have only two deterministic choices — “stay” or “move” — while the model is bilaterally based. Actual global migration is more complex. Individuals are not only faced with a yes or no decision but also must choose the optimaldestination from various potential destination countries/regions. The reality of global migration requires a new probabilistic decision model.

This paper attempts to compensate to a certain extent for the two insufficiencies described from the perspective of statistical physics. Recently, physicists have attempted to analyse human mobility with statistical methods and models and have obtained significant results^35^,^36^,^37^,^38^,^39^,^40^,^41^,^42^,^43^. Although these studies have not focused on macro global migrant flows and barriers, the application of physics-based ideas and models inspires our work. In fact, from the perspective of statistical physics, the global migration pattern has emerged from a significant number of individual behaviours based on migration preferences and conditions, including cost, which is better to discuss from an interdisciplinary perspective. Based on the classical self-selection theory, we extend the individual relocation choice to a probabilistic decision by introducing the Boltzmann factor, and set up a multilateral migration model to analyse the global migrant phenomenon. The paper is organized as follows: In Section 2, we introduce the data source and our multilateral migration model. Section 3 is an empirical analysis of global migration among 153 countries/regions. In addition to visualization of the global pattern of international migration, the migration costs are estimated and some interesting facts are revealed. Additionally, we attempt to deconstruct the barriers to international mobility using a regression analysis. Section 4 provides the conclusions and discussion.

## Materials and Methods

### Data Sources

Aside from visualization of global migration patterns, we used the data source to calculate the migration costs and process the regression analysis. A full description of the data source is in Table 1. The cross-country migration data used in this paper are from the World Bank. Based on the availability and integrity of the data, we selected 153 countries/regions according to their classification. The 153 countries/regions selected include 46 high income, 40 upper middle income, 39 lower middle income, and 28 lower income countries/regions, and these are distributed in the following way: 23 East Asian and Pacific, 46 European and Central Asian, 27 Latin American & Caribbean, 10 Middle Eastern & North African, 2 North American, 5 South Asian, and 40 sub-Saharan African countries (Table S1).

The differences between the bilateral estimates of migrant stocks in 2000 and 2010 describe the amount of migration flow. We used the population and the approximate GNI per capita as the average income of native workers. Suppose that the incomes follow a simplistic Gamma distribution, we can then fit the standard deviation from the Gini coefficients and allow it to describe the wage differentials. The details of the method will be shown in the next section.

### Migration Costs and Multilateral Migration Model

In the self-selection theory, Borjas abstracts all other economic data, except earnings, and all non-economic aspects as the migration cost *C*. He indicates that the individual moving choice is determined by the sign of the index function as follows:


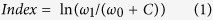


Here, *ω*_0_ and *ω*_1_ are the earnings of native workers in their countries of origin and the potential destination country. When *Index* > 0, the individual will choose to emigrate from country 0 to country 1; otherwise, he or she will stay in country 0. The individual moving choice depends on the sign of *ω*_1_ − *ω*_0_ − *C*.

Inspired by this idea, we define the net benefit for an individual *k* migrating from country *i* (the “countries” in this paper denote “countries/regions” if there is no specific explanation), given that *j* is the candidate destination country, as equal to the difference between the wage differentials and migration costs as follows:


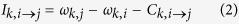


Here, *ω*_*k, i*_ and *ω*_*k, j*_ are agent *k*′*s* possible income in countries *i* and *j. C*_*k, i*→*j*_ is the migration cost expect wage difference from country *i* to *j* and *C*_*k, i*→*i*_ = 0.

Contrary to Roy’s and Borjas’ ideas, we think that the decision to migrate cannot be simply described with only “yes” or “no”, especially when individuals have the option of multiple potential destination countries. In our model, the decision to migrate to any destination is probabilistic, and we introduced the Boltzmann factor, which stems from statistical physics but is widely used to describe the decision probability when facing multiple options^44^.

Formally, suppose there are *N* countries for an individual *k* in origin country *i*. The individual has multiple potential destination countries *j* = 1, 2, …. *N*. When considering country *j* as the potential destination, based on the net benefits *I*_*k, i*→*j*_, the probability of choosing country *j* is


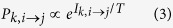


Here, *T* characterizes the individuals’ rationality. For example, the case of *T* = 0 corresponds to absolute rationality, where the migration probability is 0 if *I*_*k, i*→*j*_ < 0 and 1 if *I*_*k, i*→*j*_ > 0. *T* → ∞ corresponds to completely random decision making. *T* and *I* have the same dimension.

At the same time, *P*_*k, i*→*i*_ is the probability of an individual *k* staying in country *i*. Through the normalization of 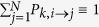, we obtain


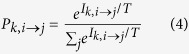


Then, the average probability of all people in country *i* deciding to migrate to *j* is


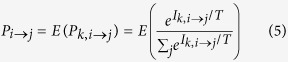


Here, *E* (·) represents the expectation function for each individual *k*.

Based on the preference function *P*_*k,i→j*_ for an individual *k* in origin country *i,* the expected proportion of the population that moves to country *j*, the potential migrants, versus those staying in country *i* is shown in Equation (6) below.


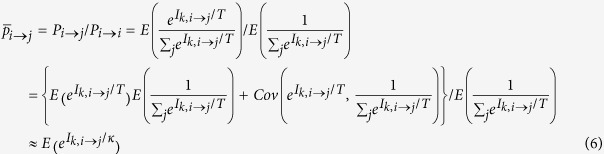


Here, *Cov* (·) is the covariance function. We suppose that the net benefits from migrants to different countries are independent of one another, and there is no strong linear correlation between 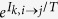 and 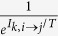. Assuming the independence of *μ*_*i*_, *μ*_*j*_ and *C*_*k, i*→*j*_, and by including equation (2), we obtain


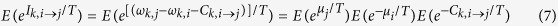


We suppose that potential migrants with the same origin and destination countries face equal migration costs under comparable circumstances, i.e., 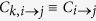. Furthermore, we suppose that the incomes of different countries adhere to a Gamma distribution^45^,^46^, i.e., 
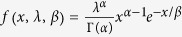
 for *x* ≥ 0, *α* > 0, *λ* > 0. By combining equations (6) and (7) and including the definition of moment-generating function and integral computation^47^, we obtain


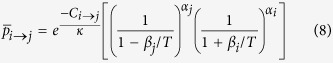


Finally, we obtain the dimensionless barrier index as


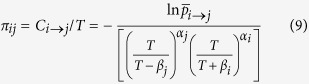


We can therefore calculate the migration costs based on real data and an appropriate *T*. The migration cost matrix {*π*_*ij*_} is non-dimensional.

## Results

### Distribution of Net Immigration

We define the net immigration for one country as the difference between immigrant flows from other countries and emigrant flows to other countries. If *n*_*i*→*j*_ is the migration flow from country *i* to *j*, then 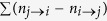 stands for the net immigration of country *i*.

**Figure 1:**
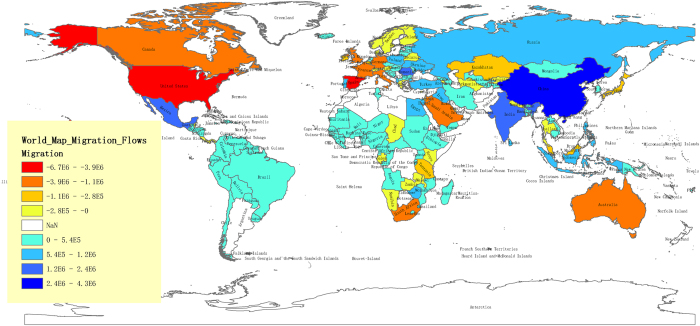
Net International Migration Flow Map. The colours describe the net international migration. Blue indicates net emigration countries/regions, and red indicates the net immigration countries/regions. Empty spaces indicate countries/regions with missing data. The maps were generated using ArcGIS 10.2 (www.esri.com/software/arcgis).

Figure 1 shows a heat map of net immigration. The colour represents the amount of net immigration. Blue indicates negative net immigration, and red describes net positive immigration. The U.S., Spain, France, Germany, Saudi Arabia, Hong Kong, Canada, Italy, Jordan, and the United Kingdom (U.K.) have the greatest amount of net immigration, which means that many people choose to migrate to these countries and that the emigrant out-flows are lower. By contrast, China, India, Mexico, Romania, Turkey, Russia, Philippines, Egypt, Poland, and Zimbabwe are the ten lowest-ranked countries for immigration.

**Figure 2:**
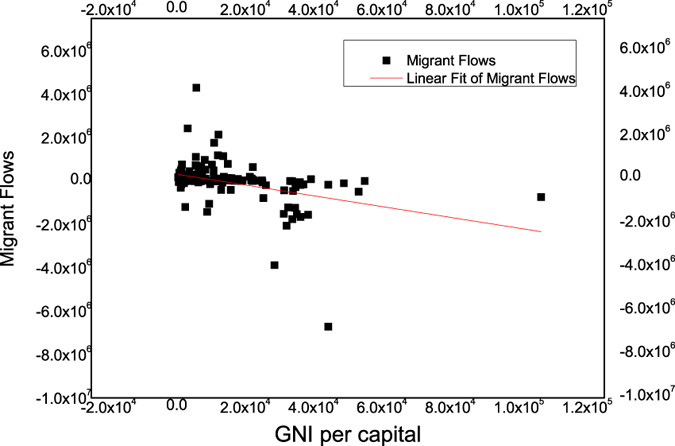
Correlation Analysis of GNI and Migrant Flows. Significant negative correlation between GNI per capita and Migrant Flows. Number: 153; *P* = 0.01; Correlation coefficient: −0.396**; *Adj. R*-square: 0.15083.

Intuitively, the share of net immigration is higher for developed countries, and, conversely, the proportion of net immigration is lower for developing countries. We are able to describe the characteristics quantitatively. A country’s development level can be represented by the indicator of GNI per capita. We find that GNI per capita and migrant flows have a significantly negative correlation among the 153 countries, and the correlation coefficient is −0.396 (Fig. 2).

### Visualization of Migration Flows Among Groups

**Figure 3:**
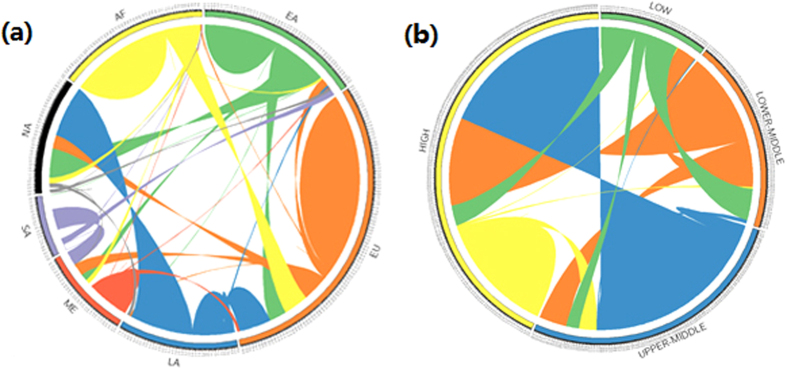
The Flows of Migration: (**a**) EA: East Asian and Pacific countries. EU: European and Central Asian countries. LA: Latin Americaan and Caribbean countries. ME: Middle Eastern and North African countries. SA: South Asian countries. NA: North American countries. AF: Sub-Saharan African countries. (**b**) Low: Low-income countries. Lower-Middle: Low-middle-income countries. Upper-Middle: Upper-middle-income countries. High: High-income countries.

In the discussion of international issues, researchers often merge different countries into one group according to the region or economic situation. For example, China is described both as a developing country and as a country in Asia. We are able to define migration flow between groups; the migration flow between groups *A* and *B* is calculated as 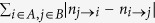, and the direction is determined by the sign of 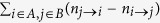. If the sign is positive, the migration flow direction is from *B* to *A*. Otherwise, the direction is from *A* to *B*. We used Circos, a software package that is widely used in genomics^48^, to show the flow of migration during the period 2000–2010. Figure 3 shows a visualization of global migration flows with regional migration flows in Fig. 3a and income states in Fig. 3b (we use the general classification of countries from the World Bank). Each classification has its own colour for the emigration flow. AF (sub-Saharan Africa), for example, is yellow, meaning that 1) there are few immigrants to this region (other colour flows at the end of the yellow arc, clockwise) compared with the sizeable emigration (yellow flows at the beginning of the yellow arc, clockwise); and 2) most emigration flows occur within the region — that is, most emigrants move to other sub-Saharan Africa countries.

Figure 3a illustrates that 1) the highest intra-regional flows are in “Europe and Central Asia” (12,457,364); 2) the highest inter-regional flows are from “Latin America and the Caribbean” to “North America”, (4,298,293); 3) “Latin America and the Caribbean”, “East Asia and the Pacific”, “sub-Saharan Africa”, and “South Asia” are clear regions of emigration, and the highest emigration flows, in descending order, are “Latin America-North America”, “East Asia-North America”, “sub-Saharan Africa-Europe and Central Asia”, and “South Asia-Middle East and North Africa”; and 4) “North America” is a distinctive migration target, with most migrants from “Latin America and the Caribbean”, “East Asia and the Pacific”, and “Europe and Central Asia”.

Figure 3b illustrates that 1) the highest intra-category flow is in high-income countries (9,295,801); other groups have relatively fewer intra-category migrants; 2) the highest inter-category flow is from uppermiddle to high-income countries (15,424,889); most migrants move to countries with higher incomes, although there are some individuals who move to poorer countries; and 3) High income countries are obvious immigration clusters.

### Migration Barrier Matrix

To calculate the migration costs in Equation (9), we need to confirm the appropriate *T* in advance. Because we suppose that the incomes in different countries adhere to a Gamma distribution as 
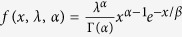
 for *x* ≥ 0, *α *> 0, *λ* > 0 and that Γ (·) is a gamma function, the expectation of the Gamma-distributed data are *E*(*x*) = *αβ*, and the Gini coefficient is 
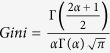
49. We use the average incomes and Gini coefficient to calculate the estimated value of parameters *α* and *β*, and we are then able to obtain the variance of incomes. Finally, the migration costs can be quantified with equation (9). In addition, we suppose that the differences in international migration costs will decrease as global integration increases. Here, our optimization goal is the minimum standard deviation for migrants and the corresponding *T* = 3.5 × 10^4^.

**Figure 4:**
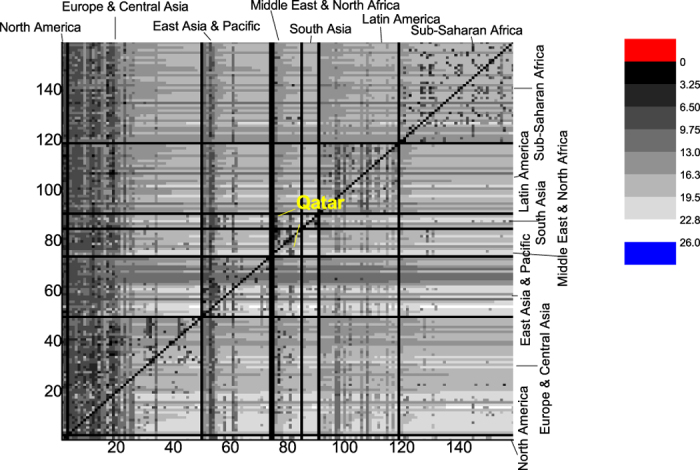
Migration Cost matrix, ordered by region. The order of the regions is “North America”, “Europe and Central Asia”, “East Asia and Pacific”, “Middle East and North Africa”, “South Asia”, “Latin America and the Caribbean”, “Sub-Saharan Africa”, from left to right on the horizontal axis and from low to high on the vertical axis.

The estimation of the migration cost matrix {*π*_*ij*_} (Table S2) by Equation (9) is shown in Fig. 4. The Matrix is asymmetrical. In Fig. 4, the matrix element of *i, j* denotes the migration cost that individuals face when moving from country *i* to country *j*.

Figure 4 exhibits the migration cost matrix ordered by region. Black indicates a lower mobility cost between two countries, and cyan denotes a higher mobility cost. Countries in the same region are in descending order of GNI per capita. 1) Qatar is a unique country with the lowest migration costs for emigrants and the highest costs for immigrants; 2) viewed as a whole, the intra-regional costs are lower than the inter-regional ones; 3) In “Europe and Central Asia”, most countries with higher GNI per capita have lower migration costs for immigrants; 4) for a specific country of origin, emigrants to different destinations face similar costs, and immigrants from different origins have similar costs. The data are presented using horizontal and vertical stripes.

### Typical Immigration and Emigration Countries: U.S. and China

**Figure 5:**
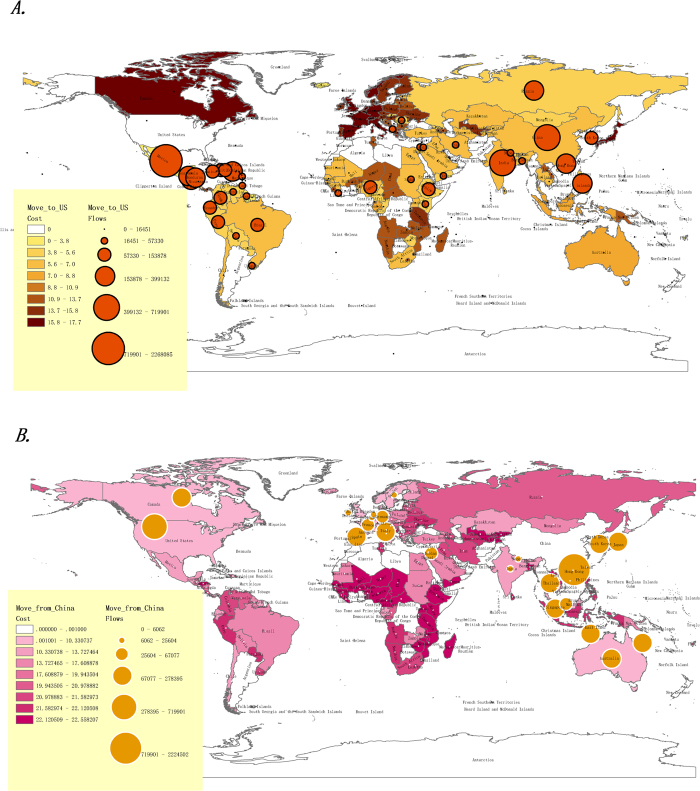
Typical immigration and emigration countries: The U.S. and China. (**A**) The U.S. had the greatest amount of immigration during 2000–2010. The size of the circle denotes the net immigration from other origin countries. The colour of the origin country shows the migration costs for immigrants, with dark colours representing high costs and light colours representing low costs from origin countries to the U.S. (**B**) China had the highest emigration level during 2000–2010. The size of the circle denotes the net emigration from China to other destination countries. The colour of the destination country shows the migration costs for emigrants, with dark colours representing high costs and light colours representing low costs to other countries. Maps were generated using ArcGIS 10.2 (www.esri.com/software/arcgis).

From 2000 to 2010, the U.S. had the highest immigration and China had the highest emigration. Figure 5 shows the distribution of migration costs for the U.S., a typical immigration country (Fig. 5A), and for China, a typical emigration country (Fig. 5B).

In Fig. 5, the size of the circle denotes the net immigrant/emigrant flows. The colour of the origin country shows the migration costs for immigrants and emigrants, with dark colours representing high costs and light colours representing low costs. The results indicate the distinctive regional characteristics.

Table 2 lists the ten origin countries with lowest and highest costs for immigrants to the U.S. and the ten destination countries with lowest and highest costs for emigrants from China.

### Correlations Between the Migration Costs for Emigrants and Immigrants

For country *i*, we use 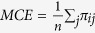 (average migration costs for emigration from country *i*) and 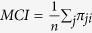 (average migration costs for immigration to country *i*) to distinguish the costs for emigrants and immigrants. The 20 countries with the lowest and highest average migration costs are listed in Table 3.

**Figure 6:**
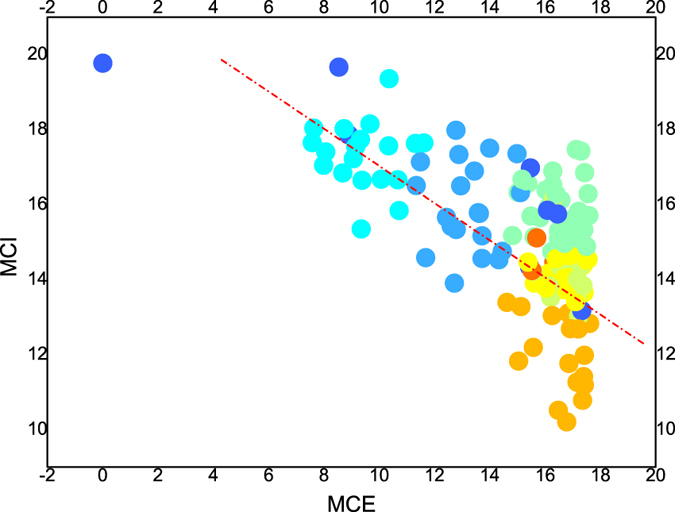
Correlations between migration costs in the origin and destination countries. The chart shows a significant and negative correlation with the coefficient as −0.329**(P = 0.01). The different colours present nine clusters in section 5.

Table 3 shows some overlap in the columns for *MCE* smallest and *MCI* largest. For example, Qatar has the lowest *MCE* and the highest *MCI*, and there is more than 50% overlap between the top 20 countries with the lowest *MCE* and those with the highest *MCI*. The phenomenon implies a negative relationship between *MCE* and *MCI*. Figure 6 describes a negative correlation between each pair of *π*_*ij*_ and *π*_*ji*_ for the 153 countries. The Pearson coefficient is −0.329 (P = 0.01).

For the source country, *π*_*ij*_ represents the non-income migration barriers that latent emigrants encounter. For the destination country, *π*_*ji*_ represents the non-income migration barriers that immigrants must overcome. Thus, a negative correlation between the mobility obstacles for emigrants and immigrants is clear. It is easy to understand that destination countries with higher entrance requirements are less attractive to individuals and that the residents are more inclined to emigrate, which will decrease the migration costs for emigrants. In addition, because the migration costs are influenced by some asymmetrical factors, such as migration policy, the correlation is complicated, and it is difficult to obtain a linear fit.

### Cluster Analysis of Countries by Migration Cost Distribution

In this paper, we conduct a cluster analysis of migration cost distribution and attempt to classify the 153 countries and describe the characteristics of the different categories. In this paper, each country has a 306-dimensional vector, with 153 migration costs for emigrants and 153 for immigrants (including the emigrants from and immigrants to the same countries). We cluster the countries with a high-dimensional vector using *k*-means clustering.

**Figure 7:**
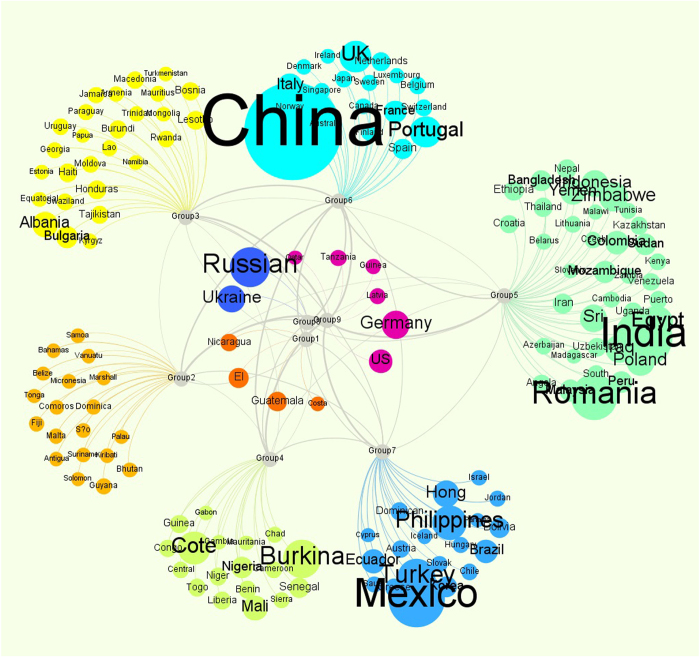
The clustering of 153 countries. 1) The clustering is based on a 306-dimensional vector, with 153 MCE and 153 MCI for each country/region. 2) used k-means clustering. 3) nodes represent the source countries. The size of the nodes indicates the migration flows from the source countries. The edges represent the distance from the countries to the k-cores and between different cores. 4) The graph shows the clustering through colours with nine groups of countries.

**Figure 8:**
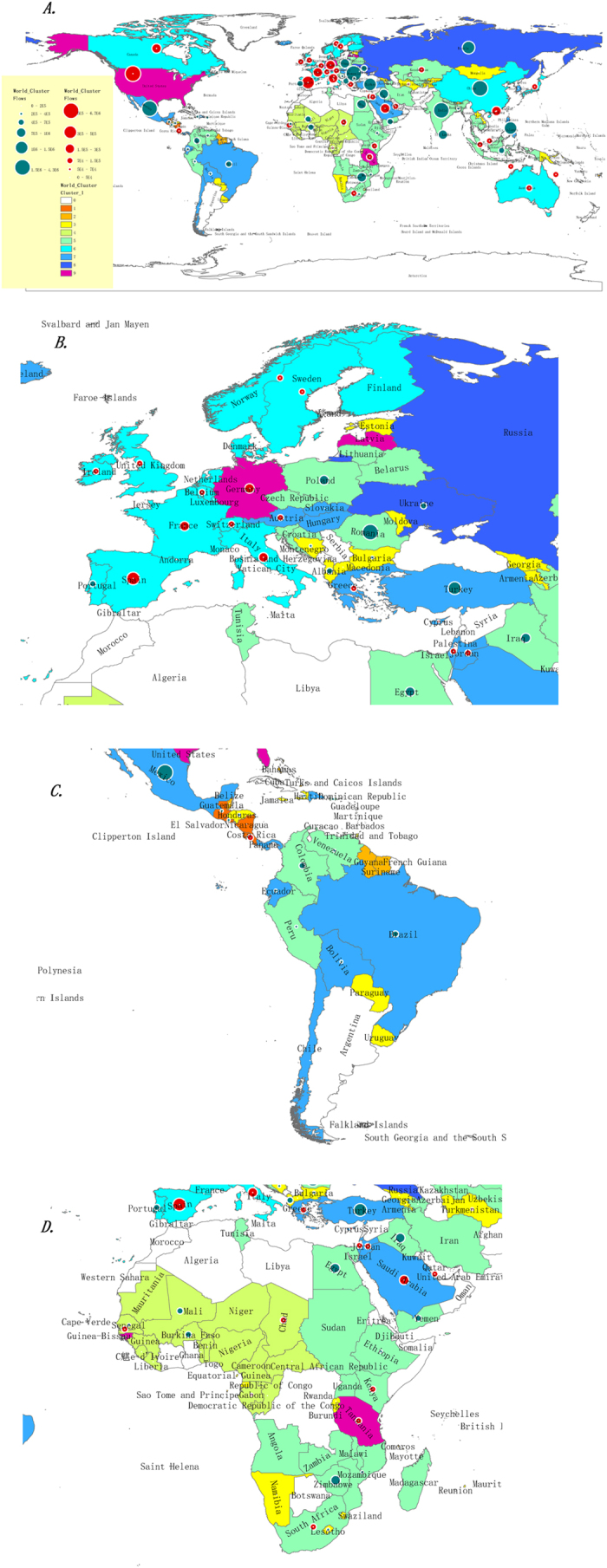
World Map of MC clustering. There are 9 categories (Group 0 indicates countries with missing data). (**A**) World map. The size of the circles denotes the quantity of migration. Green denotes the emigration countries, and red denotes the immigration countries. (**B**) European map with sub-categories. (**C**) Latin-American map with sub-categories. (**D**) African map with sub-categories. The maps were generated using ArcGIS 10.2 (www.esri.com/software/arcgis).

We classify the 153 countries into nine groups, as listed in Table S1 and shown in Figs 7 and 8. Countries in the same group have similar migration cost distribution patterns. Presentation of the clustering results shows obvious regional and economic characteristics.

Table 4 and Fig. 8 show the regional and economic characteristics of the migration cost distribution.

We were able to identify some details by analysing the data from a geographic and economic perspective. First, in terms of geography,

(1) Most “sub-Saharan Africa” countries belong to Groups 3, 4 and 5, and the distribution of these countries shows the typical regional characteristics. (Fig. 8D).

(2) The cluster of “Latin America & Caribbean” shows the regional characteristics. The countries in Group 1 are all in or around the Caribbean. Groups 7 and 5 cover more than half of the area, and the countries in Group 5 are in the northwest.

(3) Most “Western European” countries are in Group 6, and most “Central and Eastern European” countries are in Groups 5 and 3. Russia and Ukraine are the only two countries in Group 8.

(4) Except Bhutan, the other “South Asia” countries are all in Group 5.

(5) Most “Middle East & North Africa” countries are in Group 5 and 7.

Second, in terms of economics,

(1) Over 60% of High Income countries are in Groups 6 and 7.

(2) 75% of Low Income countries are in Groups 4 and 5.

(3) Other groups do not have obvious economic characteristics.

Group 9 includes six countries with unique migration patterns. These countries alone were included in the cluster analysis. For simplicity, we put them in one group to mark their special characteristics, including Qatar, the United States, Germany, Latvia, Guinea-Bissau and Tanzania.

For example, Qatar has unique migration patterns that are different from other countries. Qatar is a high-income country in the Middle Eastern and North African region. The population rank is 131, and the GNI rank per capita is one. Qatar has the lowest MCE and highest MCI values, and the difference is quite significant. During the period 2000–2010, Qatar had 6,866 emigrants and 738,904 immigrants, with a ratio of 1:107.6. Although Qatar has the highest GNI of the 153 countries, under the premise of not considering income factors, Qatar is still the most attractive country for immigrants, and immigrants choose to pay high migration costs to settle there.

### Regression Analysis

It is meaningful to explore national migration restrictions. The regional and economic characteristics of the clusters presented in the previous section prompted us to find the factors affecting global mobility. It is clear that migration restrictions are the result of complicated and integrated factors, and we do not intend to find all these restrictions in this paper. Thus, we used data for distance, language, human development, corruption perception, economic development, environment, trade barter and migrant scale, as described in Table 1, to study the non-income factors affecting migrant mobility.

This paper uses a multiple linear regression to analyse *π*_*ij*_ for each migration pair {*i, j*} with the origin country *i* and destination country *j*. The results are in Table 5.

Several years ago, some researchers believed that the most important obstacle for immigrants is language disparity; language proficiency has a sizeable effect on earnings of up to 40%^50^,^51^. Our regression analysis shows the non-significance of language advantages for migrants for these ten years, and this seems to be a reasonable results in this globalization wave.

Munshi analyses the effects of Mexican immigrant networks and verifies that the established migrant community will help new immigrants find employment and hold higher paying non-agricultural jobs^52^. This paper indicates that the migrant scale has a negative effect on migrant restrictions and conforms to the economies of scale in immigrants, which is in line with the effect on migrant networks.

Moreover, we performed some analyses as follows:

(1) Geographic distance, GNI per capita and the PM2.5 index of the origin country have no significant effect on migrant mobility.

(2) Language has no significant effect on migrant mobility.

(3) Potential emigrants in origin countries with lower human development levels(HDI) will face higher movement restrictions; immigrants face fewer restrictions when moving to destination countries with lower human development levels.

(4) Corruption perception in destination countries has a negative effect on immigrant movement costs. Corruption perception in origin countries has a positive effect on emigrant movement costs.

(5) Potential immigrants face lower restrictions when moving to destination countries with lower GNI per capita.

(6) The migrant scale has a negative effect on migrant restrictions, which means that the more migrants there are from country *i* to *j*, the lower the migration costs are for potential emigrants.

(7) Higher pollution in destination countries increases immigrants’ movement restrictions.

(8) Trade bartering will increase the migrants’ restrictions, both for the origin and destination countries.

(9) The growth of GDP in the origin countries has a positive effect on emigrant moving costs, and the growth in destination countries has a negative effect on immigrant moving costs.

## Conclusion and Discussion

Migration costs are the basis of various migration issues, but they are difficult to quantify. This paper presents a multilateral migration model to quantify and analyse migration costs worldwide. We calculate the international migration costs based on data for migrant flows and other economic or non-economic indicators.

The data show that the migration cost matrix is asymmetrical. Given origin country *i* and destination country *j*, we find a negative linear correlation between the mobility obstacles for emigrants and immigrants. It is easy to understand that destination countries with higher entrance obstacles are less attractive to individuals and that the residents are more inclined to emigrate, which will decrease the migration costs for emigrants.

We conduct a cluster analysis of 153 countries/regions, with the vector of migration costs using *k*-means clustering, and this analysis results in nine categories. For the countries in “Sub-Sahara African”, “Latin America & Caribbean”, “Western European” and “Central & Eastern European”, the presence of clustering reveals some obvious regional and economic characteristics. It is interesting that 60% of high-income countries and 75% of low-income countries are classified into two groups separately. Seventy-three percent of sub-Saharan-Africa has been clustered into two groups with clear regional positions in the eastern and western part of the African continent. “Western European” and “Central and Eastern European” countries also have different migration patterns.

In addition, this paper attempts to confirm some of the factors affecting global mobility restrictions. Using regression analysis, we indicate the non-significance of language advantages for migrants for these ten years and we find that the migrant scale has a negative effect on migrant barriers, which conforms to the economies of scale in immigrants and are in line with the effect on migrant networks. The results are acceptable and verify the framework of the multilateral migrant model to a certain extent. However, the small *R*^*2*^ indicates that there are many more important factors affecting the movement that need to be analysed — for example, policy and cultural factors. However, these are difficult to quantify precisely and will be the focus of attention in our future studies.

## Additional Information

**How to cite this article**: Li, X. *et al*. Characterizing the International Migration Barriers with a Probabilistic Multilateral Migration Model. *Sci. Rep.* **6**, 32522; doi: 10.1038/srep32522 (2016).

## Supplementary Material

Supplementary Information

Supplementary Dataset

## Figures and Tables

**Table 1:** Description of the Data Sources.

Content	Indicator	Indicator Description	Data Source
Migration Costs	Migrant Flows	Estimates of Migrant Stocks 2000, 2010	http://www.worldbank.org/
	Migrant FlowsFor China	China only has total Migrant Stocks in the World Bank’s databaseChina’s Bilateral Flow Data are from the 2010 Population Census of the People’s Republic of China	http://www.stats.gov.cn/
	Population	Population (total), 2000–2010, Average	http://www.worldbank.org/
	Average Incomes	GNI per capita, PPP (current international $), 2000–2010, Average	http://www.worldbank.org/
	Wage Differentials	Calculated based on the Data for the Gini coefficient, 2000–2010, Average	The data of the Gini coefficient is from http://www.worldbank.org/
Regression Analysis	Distance	Geographical Distance between Capitals	http://www.baidu.com
	Language	Chiswick and Miller list the Linguistic Distance of different languages to English. We use the distance between the language of the origin and destination countries.	Barry R. Chiswick & Paul W. Miller (2005)
	Human Development Index	Human Development Index. By the United Nations, 2000, 2005, 2008, 2010, Average	http://hdr.undp.org/en/data
	Corruption Perception Index	Corruption Perception Index from Transparency International, 2009, 2010, Average	https://www.transparency.org/
	GNI	GNI per capita, PPP (current international $), 2000–2010, Average	http://www.worldbank.org/
	GDP growth	GDP growth (annual%), 2000–2010, Average	http://www.worldbank.org/
	Migrant Scale	Estimates of Migrant Stocks, 2000, 2010	http://www.worldbank.org/
	PM2.5	PM2.5 pollution, mean annual exposure (micrograms per cubic metre), 2005, 2010, Average	http://www.worldbank.org/
	Trade Barter	Net barter terms of the trade index (2000 = 100) 2000–2010, Average	http://www.worldbank.org/

**Table 2:** Origin countries with the lowest and highest costs for immigrants to the U.S. and destination countries with the lowest and highest costs for emigrants from China.

	Lowest Costs to the U.S.	Highest Costs to the U.S.	Lowest Costs from China	Highest Costs from China
1	Dominica	Japan	Qatar	Liberia
2	Micronesia	Germany	Hong Kong	Malawi
3	Marshall Islands	United Kingdom	United States	Burundi
4	Guyana	France	Singapore	Mozambique
5	El Salvador	Italy	Japan	Central African Republic
6	Puerto Rico	Canada	Canada	Ethiopia
7	Honduras	Netherlands	South Korea	Niger
8	Jamaica	Spain	Australia	Rwanda
9	Samoa	Switzerland	Italy	Uganda
10	Iceland	Norway	Spain	Guinea

**Table 3:** The countries with the lowest and highest average migration costs when ranked by MCE and MCI.

	MCE Lowest	MCI Lowest	MCE Highest	MCI Highest
1	Qatar	Dominica	Comoros	Qatar
2	Canada	Palau	Madagascar	United States
3	Spain	Marshall Islands	Ethiopia	Japan
4	Australia	Kiribati	Burundi	Italy
5	United Kingdom	Tonga	Tajikistan	Spain
6	United States	Sao Tome and Principe	Zimbabwe	Norway
7	Belgium	Micronesia	Laos	Saudi Arabia
8	Norway	Samoa	Bangladesh	Germany
9	Germany	Antigua and Barbuda	Kiribati	Netherlands
10	Sweden	Vanuatu	Vanuatu	Canada
11	Switzerland	Belize	Lesotho	China
12	Netherlands	Solomon Islands	Malawi	Singapore
13	Luxembourg	Guyana	Micronesia	France
14	Ireland	Comoros	Honduras	Switzerland
15	Italy	Bhutan	Togo	South Korea
16	Denmark	Gambia	Marshall Islands	Indonesia
17	France	Suriname	Rwanda	India
18	Japan	Fiji	Guinea-Bissau	United Kingdom
19	Finland	Guinea-Bissau	Moldova	Brazil
20	Portugal	Malta	Papua New Guinea	Mexico

**Table 4:** The Regional and Economic Characteristics of the Migration Cost Distribution.

Group	Num	Income				Region						
		High	Upper	Lower	Low	EA	EU	LA	ME	NA	SA	AF
1	4	0	1	**3**	0	0	0	**4**	0	0	0	0
2	19	3	**7**	**8**	1	9	0	**6**	1	0	1	2
3	27	4	**8**	**11**	4	3	**11**	**6**	0	0	0	7
4	18	0	1	**6**	**11**	0	0	0	0	0	0	**18**
5	38	6	**14**	**8**	**10**	4	**10**	4	**5**	0	**4**	**11**
6	19	**18**	1	0	0	4	**14**	0	0	1	0	0
7	20	**10**	**8**	2	0	3	**7**	**7**	3	0	0	0
8	2	1	0	1	0	0	2	0	0	0	0	0
9	6	4	0	0	2	0	2	0	1	1	0	2
total	153	46	40	39	28	23	46	27	10	2	5	40

A) EA: East Asian and Pacific countries. EU: European and Central Asian countries. LA: Latin American and Caribbean countries. ME: Middle Eastern and North African countries. SA: South Asian countries. NA: North American countries. AF: Sub-Saharan African countries. B) Low: Low-income countries. Lower: Low-middle-income countries. Upper: Upper-middle-income countries. High: High-income countries.

**Table 5:** Results from a Regression Analysis for Migration Costs.

Factors	Coefficient	Error	t	Sig.
(constance)	16.18833***	0.35704	45.34085	0
Distance	−6.59E-10	1.46E-08	−0.04509	9.64E-01
Language	−0.03494	0.03644	−0.95883	0.33766
HumanDevelopmentIndex(s)	−2.59169***	0.28684	−9.03546	0
HumanDevelopmentIndex(d)	9.34E-01**	0.28696	3.25488	0.00114
CorruptionPerceptionIndex(s)	0.22406***	0.02519	8.89483	0.00E + 00
CorruptionPerceptionIndex(d)	−0.42024***	0.02521	−16.67074	0
GNIper capita(s)	6.09E-07	3.59E-06	0.1693	0.86556
GNIper capita(d)	2.85E-05***	3.59E-06	7.93084	2.22E-15
GDP% (Growth Rates) (s)	0.01598***	0.00395	4.04159	5.33E-05
GDP% (Growth Rates) (d)	−0.13262***	0.01186	−11.17827	0
Migrant Scale	−28.61589***	0.66739	−42.87743	0
PM2.5(s)	5.97E-04	0.00255	0.23435	0.81472
PM2.5(d)	1.10E-02***	2.55E-03	4.32491	1.53E-05
Tradebarter(s)	3.87E-03*	1.70E-03	2.28072	2.26E-02
Tradebarter(d)	0.00401*	0.0017	2.36367	0.01811

Adj. R^2^ = 0.12524, P = 0.01, Number = 17292. (d) are the results for destination countries, (s) are the results for source or origin countries.
