# Supplementary material for: Characterizing the International Migration Barriers with a Probabilistic Multilateral Migration Model
Source: Sci Rep. 2016 Sep 6;6:32522. doi: 10.1038/srep32522 (PMC5011646; doi:10.1038/srep32522)
Supplement: Supplementary Information [file srep32522-s1.doc]

**Characterizing the International Migration Barriers with a Probabilistic Multilateral Migration Model**

Xiaomeng Li^1^, Hongzhong Xu^1^, Jiawei Chen^1^, Qinghua Chen^1^*, Jiang Zhang^1^, Zengru Di^1^*

1. School of Systems Science, Beijing Normal University

*Corresponding author: qinghuachen@bnu.edu.cn, zdi@bnu.edu.cn

**Table S1.** Description of the Data Sources

| **Content** | **Indicator** | **Indicator Description** | **Data Source** |
| --- | --- | --- | --- |
| **Migration**  **Costs** | Migrant Flows | Estimates of Migrant Stocks 2000, 2010 | http://www.worldbank.org/ |
|  | Migrant Flows  For China | China only has total Migrant Stocks in the World Bank’s database  China’s Bilateral Flow Data are from the 2010 Population Census of the People’s Republic of China | http://www.stats.gov.cn/ |
|  | Population | Population (total), 2000-2010, Average | http://www.worldbank.org/ |
|  | Average Incomes | GNI per capita, PPP  (current international $), 2000-2010, Average | http://www.worldbank.org/ |
|  | Wage Differentials | Calculated based on the Data for the Gini coefficient, 2000-2010, Average | The data of the Gini coefficient is from  http://www.worldbank.org/ |
| **Regression**  **Analysis** | Distance | Geographical Distance between Capitals | http://www.baidu.com |
|  | Language | Chiswick and Miller list the Linguistic Distance of different languages to English. We use the distance between the language of the origin and destination countries. | Barry R. Chiswick & Paul W. Miller (2005) |
|  | Human Development Index | Human Development Index  By the United Nations, 2000, 2005, 2008, 2010, Average | http://hdr.undp.org/en/data |
|  | Corruption Perception Index | Corruption Perception Index from Transparency International, 2009, 2010, Average | https://www.transparency.org/ |
|  | GNI | GNI per capita, PPP  (current international $), 2000-2010, Average | http://www.worldbank.org/ |
|  | GDP growth | GDP growth (annual %), 2000-2010, Average | http://www.worldbank.org/ |
|  | Migrant Scale | Estimates of Migrant Stocks, 2000, 2010 | http://www.worldbank.org/ |
|  | PM2.5 | PM2.5 pollution, mean annual exposure (micrograms per cubic metre), 2005, 2010, Average | http://www.worldbank.org/ |
|  | Trade Barter | Net barter terms of the trade index (2000=100)  2000-2010, Average | http://www.worldbank.org/ |

**Table S2**. The description of 153 countries and regions.

|  | **Country** | **Incomes Level** | **Region** |
| --- | --- | --- | --- |
| **1** | Albania | Upper middle income | Europe & Central Asia |
| **2** | Angola | Upper middle income | Sub-Saharan Africa |
| **3** | Antigua and Barbuda | High income | Latin America & Caribbean |
| **4** | Armenia | Lower middle income | Europe & Central Asia |
| **5** | Australia | High income | East Asia & Pacific |
| **6** | Austria | High income | Europe & Central Asia |
| **7** | Azerbaijan | Upper middle income | Europe & Central Asia |
| **8** | Bahamas, The | High income | Latin America & Caribbean |
| **9** | Bangladesh | Low income | South Asia |
| **10** | Belarus | Upper middle income | Europe & Central Asia |
| **11** | Belgium | High income | Europe & Central Asia |
| **12** | Belize | Upper middle income | Latin America & Caribbean |
| **13** | Benin | Low income | Sub-Saharan Africa |
| **14** | Bhutan | Lower middle income | South Asia |
| **15** | Bolivia | Lower middle income | Latin America & Caribbean |
| **16** | Bosnia and Herzegovina | Upper middle income | Europe & Central Asia |
| **17** | Brazil | Upper middle income | Latin America & Caribbean |
| **18** | Bulgaria | Upper middle income | Europe & Central Asia |
| **19** | Burkina Faso | Low income | Sub-Saharan Africa |
| **20** | Burundi | Low income | Sub-Saharan Africa |
| **21** | Cambodia | Low income | East Asia & Pacific |
| **22** | Cameroon | Lower middle income | Sub-Saharan Africa |
| **23** | Canada | High income | North America |
| **24** | Central African Republic | Low income | Sub-Saharan Africa |
| **25** | Chad | Low income | Sub-Saharan Africa |
| **26** | Chile | High income | Latin America & Caribbean |
| **27** | Colombia | Upper middle income | Latin America & Caribbean |
| **28** | Comoros | Low income | Sub-Saharan Africa |
| **29** | Congo, Rep. | Lower middle income | Sub-Saharan Africa |
| **30** | Costa Rica | Upper middle income | Latin America & Caribbean |
| **31** | Côte d'Ivoire | Lower middle income | Sub-Saharan Africa |
| **32** | Croatia | High income | Europe & Central Asia |
| **33** | Cyprus | High income | Europe & Central Asia |
| **34** | Czech Republic | High income | Europe & Central Asia |
| **35** | Denmark | High income | Europe & Central Asia |
| **36** | Dominica | Upper middle income | Latin America & Caribbean |
| **37** | Dominican Republic | Upper middle income | Latin America & Caribbean |
| **38** | Ecuador | Upper middle income | Latin America & Caribbean |
| **39** | Egypt, Arab Rep. | Lower middle income | Middle East & North Africa |
| **40** | El Salvador | Lower middle income | Latin America & Caribbean |
| **41** | Equatorial Guinea | High income | Sub-Saharan Africa |
| **42** | Estonia | High income | Europe & Central Asia |
| **43** | Ethiopia | Low income | Sub-Saharan Africa |
| **44** | Fiji | Upper middle income | East Asia & Pacific |
| **45** | Finland | High income | Europe & Central Asia |
| **46** | France | High income | Europe & Central Asia |
| **47** | Gabon | Upper middle income | Sub-Saharan Africa |
| **48** | Gambia, The | Low income | Sub-Saharan Africa |
| **49** | Georgia | Lower middle income | Europe & Central Asia |
| **50** | Germany | High income | Europe & Central Asia |
| **51** | Greece | High income | Europe & Central Asia |
| **52** | Guatemala | Lower middle income | Latin America & Caribbean |
| **53** | Guinea | Low income | Sub-Saharan Africa |
| **54** | Guinea-Bissau | Low income | Sub-Saharan Africa |
| **55** | Guyana | Lower middle income | Latin America & Caribbean |
| **56** | Haiti | Low income | Latin America & Caribbean |
| **57** | Honduras | Lower middle income | Latin America & Caribbean |
| **58** | Hong Kong, China | High income | East Asia & Pacific |
| **59** | Hungary | Upper middle income | Europe & Central Asia |
| **60** | Iceland | High income | Europe & Central Asia |
| **61** | India | Lower middle income | South Asia |
| **62** | Indonesia | Lower middle income | East Asia & Pacific |
| **63** | Iran, Islamic Rep. | Upper middle income | Middle East & North Africa |
| **64** | Iraq | Upper middle income | Middle East & North Africa |
| **65** | Ireland | High income | Europe & Central Asia |
| **66** | Israel | High income | Middle East & North Africa |
| **67** | Italy | High income | Europe & Central Asia |
| **68** | Jamaica | Upper middle income | Latin America & Caribbean |
| **69** | Japan | High income | East Asia & Pacific |
| **70** | Jordan | Upper middle income | Middle East & North Africa |
| **71** | Kazakhstan | Upper middle income | Europe & Central Asia |
| **72** | Kenya | Low income | Sub-Saharan Africa |
| **73** | Kiribati | Lower middle income | East Asia & Pacific |
| **74** | Korea, Rep. | High income | East Asia & Pacific |
| **75** | Kyrgyz Republic | Lower middle income | Europe & Central Asia |
| **76** | Lao PDR | Lower middle income | East Asia & Pacific |
| 77 | Latvia | High income | Europe & Central Asia |
| 78 | Lesotho | Lower middle income | Sub-Saharan Africa |
| 79 | Liberia | Low income | Sub-Saharan Africa |
| 80 | Lithuania | High income | Europe & Central Asia |
| 81 | Luxembourg | High income | Europe & Central Asia |
| 82 | Macedonia, FYR | Upper middle income | Europe & Central Asia |
| 83 | Madagascar | Low income | Sub-Saharan Africa |
| 84 | Malawi | Low income | Sub-Saharan Africa |
| 85 | Malaysia | Upper middle income | East Asia & Pacific |
| 86 | Mali | Low income | Sub-Saharan Africa |
| 87 | Malta | High income | Middle East & North Africa |
| 88 | Marshall Islands | Upper middle income | East Asia & Pacific |
| 89 | Mauritania | Lower middle income | Sub-Saharan Africa |
| 90 | Mauritius | Upper middle income | Sub-Saharan Africa |
| 91 | Mexico | Upper middle income | Latin America & Caribbean |
| 92 | Micronesia, Fed. Sts. | Lower middle income | East Asia & Pacific |
| 93 | Moldova | Lower middle income | Europe & Central Asia |
| 94 | Mongolia | Lower middle income | East Asia & Pacific |
| 95 | Mozambique | Low income | Sub-Saharan Africa |
| 96 | Namibia | Upper middle income | Sub-Saharan Africa |
| 97 | Nepal | Low income | South Asia |
| 98 | Netherlands | High income | Europe & Central Asia |
| 99 | Nicaragua | Lower middle income | Latin America & Caribbean |
| 100 | Niger | Low income | Sub-Saharan Africa |
| 101 | Nigeria | Lower middle income | Sub-Saharan Africa |
| 102 | Norway | High income | Europe & Central Asia |
| 103 | Palau | Upper middle income | East Asia & Pacific |
| 104 | Panama | Upper middle income | Latin America & Caribbean |
| 105 | Papua New Guinea | Lower middle income | East Asia & Pacific |
| 106 | Paraguay | Lower middle income | Latin America & Caribbean |
| 107 | Peru | Upper middle income | Latin America & Caribbean |
| 108 | Philippines | Lower middle income | East Asia & Pacific |
| 109 | Poland | High income | Europe & Central Asia |
| 110 | Portugal | High income | Europe & Central Asia |
| 111 | Puerto Rico | High income | Latin America & Caribbean |
| 112 | Qatar | High income | Middle East & North Africa |
| 113 | Romania | Upper middle income | Europe & Central Asia |
| 114 | Russian Federation | High income | Europe & Central Asia |
| 115 | Rwanda | Low income | Sub-Saharan Africa |
| 116 | Samoa | Lower middle income | East Asia & Pacific |
| 117 | São Tomé and Principe | Lower middle income | Sub-Saharan Africa |
| 118 | Saudi Arabia | High income | Middle East & North Africa |
| 119 | Senegal | Lower middle income | Sub-Saharan Africa |
| 120 | Sierra Leone | Low income | Sub-Saharan Africa |
| 121 | Singapore | High income | East Asia & Pacific |
| 122 | Slovak Republic | High income | Europe & Central Asia |
| 123 | Slovenia | High income | Europe & Central Asia |
| 124 | Solomon Islands | Lower middle income | East Asia & Pacific |
| 125 | South Africa | Upper middle income | Sub-Saharan Africa |
| 126 | Spain | High income | Europe & Central Asia |
| 127 | Sri Lanka | Lower middle income | South Asia |
| 128 | Sudan | Lower middle income | Sub-Saharan Africa |
| 129 | Suriname | Upper middle income | Latin America & Caribbean |
| 130 | Swaziland | Lower middle income | Sub-Saharan Africa |
| 131 | Sweden | High income | Europe & Central Asia |
| 132 | Switzerland | High income | Europe & Central Asia |
| 133 | Tajikistan | Low income | Europe & Central Asia |
| 134 | Tanzania | Low income | Sub-Saharan Africa |
| 135 | Thailand | Upper middle income | East Asia & Pacific |
| 136 | Togo | Low income | Sub-Saharan Africa |
| 137 | Tonga | Upper middle income | East Asia & Pacific |
| 138 | Trinidad and Tobago | High income | Latin America & Caribbean |
| 139 | Tunisia | Upper middle income | Middle East & North Africa |
| 140 | Turkey | Upper middle income | Europe & Central Asia |
| 141 | Turkmenistan | Upper middle income | Europe & Central Asia |
| 142 | Uganda | Low income | Sub-Saharan Africa |
| 143 | Ukraine | Lower middle income | Europe & Central Asia |
| 144 | United Kingdom | High income | Europe & Central Asia |
| 145 | United States | High income | North America |
| 146 | Uruguay | High income | Latin America & Caribbean |
| 147 | Uzbekistan | Lower middle income | Europe & Central Asia |
| 148 | Vanuatu | Lower middle income | East Asia & Pacific |
| 149 | Venezuela, RB | Upper middle income | Latin America & Caribbean |
| 150 | Yemen, Rep. | Lower middle income | Middle East & North Africa |
| 151 | Zambia | Lower middle income | Sub-Saharan Africa |
| 152 | Zimbabwe | Low income | Sub-Saharan Africa |
| 153 | China | Upper middle income | East Asia & Pacific |

**Table S3**. The Migration Costs Matrix.

Uploaded as supplementary database.
